# Comparing Molnupiravir to Nirmatrelvir/Ritonavir (Paxlovid) in the Treatment of Mild-to-Moderate COVID-19 in Immunocompromised Cancer Patients

**DOI:** 10.3390/cancers16051055

**Published:** 2024-03-05

**Authors:** Andrea J. Haddad, Ray Y. Hachem, Mohamed Moussa, Ying Jiang, Hiba R. Dagher, Patrick Chaftari, Anne-Marie Chaftari, Issam I. Raad

**Affiliations:** 1 Department of Infectious Diseases, Infection Control and Employee Health, The University of Texas MD Anderson Cancer Center, Houston, TX 77030, USA; ajhaddad@mdanderson.org (A.J.H.); mmoussa@mdanderson.org (M.M.); yijiang@mdanderson.org (Y.J.); hrdagher@mdanderson.org (H.R.D.); achaftari@mdanderson.org (A.-M.C.); iraad@mdanderson.org (I.I.R.); 2 Department of Emergency Medicine, The University of Texas MD Anderson Cancer Center, Houston, TX 77030, USA; pchaftari@mdanderson.org

**Keywords:** COVID-19, coronaviruses, SARS-CoV-2, cancer, immunocompromised, therapy, treatment, molnupiravir, nirmatrelvir/ritonavir, paxlovid

## Abstract

**Simple Summary:**

The results of previous studies may suggest that Nirmatrelvir/Ritonavir, when evaluated against placebo controls, could potentially be more effective than Molnupiravir in reducing COVID-19 progression. While Nirmatrelvir/Ritonavir reduced COVID-19 progression risk by 88% versus placebo, Molnupiravir reduced it by 31%. However, there has been no direct head-to-head comparison between the two drugs in a prospective randomized trial. This study compared the safety and effectiveness of Nirmatrelvir/Ritonavir versus Molnupiravir in the treatment of mild-to-moderate COVID-19 in 240 immunocompromised cancer patients who received one of the two drugs. The results showed no significant differences in the rate of disease progression between the treatments. Nevertheless, those treated with Nirmatrelvir/Ritonavir reported a higher incidence of drug interactions and adverse events. Therefore, although both medications are effective in preventing severe disease or death in cancer patients with COVID-19, Molnupiravir could be safer than Nirmatrelvir/Ritonavir in terms of drug interactions and adverse effects.

**Abstract:**

Background: Nirmatrelvir/Ritonavir has been shown to reduce the risk of COVID-19 progression by 88% compared to placebo, while Molnupiravir reduced it by 31%. However, these two agents have not been compared head-to-head. We therefore compared the safety and efficacy of both agents for the treatment of mild-to-moderate COVID-19 in immunocompromised cancer patients. Methods: We identified 240 cancer patients diagnosed with COVID-19 and treated with Molnupiravir or Nirmatrelvir/Ritonavir. Patients were matched using a 1:2 ratio based on age group (18–64 years vs. ≥65) and type of cancer. The collected data included demographics, comorbidities, and treatment outcome. Results: Both groups had comparable characteristics and presenting symptoms. However, dyspnea was more prevalent in the Molnupiravir group, while sore throat was more prevalent in the Nirmatrelvir/Ritonavir group. The rate of disease progression was comparable in both groups by univariate and multivariable analysis. Treatment with Molnupiravir versus Nirmatrelvir/Ritonavir revealed no significant difference in disease progression by multivariable analysis (adjusted OR = 1.31, 95% CI: 0.56–3.14, *p* = 0.70). Patients who received Nirmatrelvir/Ritonavir, however, were significantly more prone to having drug–drug interactions/adverse events (30% vs. 0%, *p* < 0.0001). Conclusions: In the treatment of mild-to-moderate COVID-19 in cancer patients, Molnupiravir was comparable to Nirmatrelvir/Ritonavir in preventing progression to severe disease/death and rebound events, and it had a superior safety profile.

## 1. Introduction

In December 2019, the emergence of COVID-19 caused by SARS-CoV-2 in Wuhan, China, rapidly affected the whole world. The virus quickly spread globally, leading to the World Health Organization (WHO) declaring it a Public Health Emergency of International Concern on 30 January 2020, and later declaring it a pandemic on 11 March 2020. Moreover, several studies on COVID-19 showed that at-risk populations, specifically cancer patients, suffered worse outcomes [1,2,3,4,5,6,7,8]. With more than seven million deaths caused, the search for a cure was and still is at the forefront [9,10].

Vaccine developments were underway and mass vaccination campaigns took place, which resulted in significant reductions in severe disease and death [11,12,13,14]. In October 2020, the FDA approved Remdesivir as the first antiviral effective against severe SARS-CoV-2 and, while Remdesivir proved effective, it was expensive and could only be used in a hospital setting [15]. Effective oral agents for non-hospitalized patients were still needed [16]. Hence, in December 2021, the FDA issued an emergency use authorization for both Nirmatrelvir/Ritonavir (Paxlovid) and Molnupiravir as oral antivirals for patients with at least one risk factor for severe illness [17]. Phase 3 clinical studies have shown both antivirals to be effective in the treatment of COVID-19 by decreasing the progression to severe disease and by reducing mortality.

Molnupiravir is an oral antiviral medication that functions by introducing errors during viral RNA replication, hindering the virus’s ability to proliferate [18]. It has been shown to reduce the risk of hospitalization or death compared to placebo in patients with a poor prognosis and with mild COVID-19 by 31%, with minimal side effects [19]. Molnupiravir has gained considerable attention for its preferable pharmacokinetics and minimal adverse side effects [18].

Nirmatrelvir/Ritonavir, another oral antiviral medication, works by inhibiting the activity of the virus’s main protease [18]. It quickly became the first-line treatment, as it was shown to reduce the risk of hospitalization by 88% when compared to placebo in unvaccinated individuals during a delta variant wave. A study performed in an outpatient population in Quebec, Canada, during a BA.2 and BA.4/5 Omicron surge showed that in unvaccinated or incompletely vaccinated patients, the risk reduction in COVID-19-related hospitalization was 96%, while treatment of vaccinated patients with the antiviral had no significant effect on the risk of hospitalization and disease progression unless the patients were immunocompromised (transplant or neutropenic patients) or were over 70 years old [20]. A different study showed that Molnupiravir was also safe and effective among vaccinated Japanese patients with COVID-19, including the elderly and those with multiple comorbidities [21].

A study performed in Europe matched a total of 116 patients with a hematological malignancy from the EPICOVIDEHA registry who received Molnupiravir monotherapy with those who received Nirmatrelvir/Ritonavir. No statistically significant difference was found between treatment arms for hospitalization, mortality rates, or survival probability [22]. Another study performed in northern Italy followed a total of 69 patients with solid tumors on active treatment, whereby an infectious disease specialist had chosen the most appropriate drug among Sotrovimab, Molnupiravir, Remdesivir, and Nirmatrelvir/Ritonavir. They concluded that early therapies may reduce the morbidity of COVID-19 in patients with solid tumors [23].

In a study comparing the rebound of both drugs, COVID-19 rebound was found for both. Although the patients who received Molnupiravir were significantly older and had more comorbidities than patients who received Nirmatrelvir/Ritonavir, after propensity-score matching there was no significant difference in rebound or hospitalization between the two groups [24]. As stated in both agents’ labeling, viral RNA rebound post-treatment with Molnupiravir or Paxlovid is not associated with the primary clinical outcome of COVID-19-related hospitalization or death from any cause by day 28 following the single 5-day course. Remdesivir is another anti-COVID-19 antiviral agent, with several studies showing its efficacy in both hospitalized COVID-19 patients with pneumonia as well as outpatients with mild-to-moderate COVID-19 [25,26,27,28,29]. However, remdesivir is not available as an oral agent and hence there could be logistic limitations to giving it on an outpatient basis.

While there have been multiple international studies investigating the effectiveness of these two anti-COVID-19 drugs (Molnupiravir and Nirmatrelvir/Ritonavir), the results have varied. Notably, there has been no study that has compared them both in similar matched cohort arms in the United States. Several studies on COVID-19 showed that patients with cancer had poorer outcomes. There is a gap in the literature concerning the efficacy, outcomes, and safety of these drugs in an at-risk population such as an immunocompromised cancer population. The importance of this proposed study therefore lies in filling this knowledge gap and comparing the two agents in the same high-risk cancer patient population.

## 2. Patients and Methods

### 2.1. Patient Selection

This retrospective single-center study included 240 patients with a hematologic malignancy or solid tumors who were diagnosed with mild-to-moderate COVID-19 by Real-time polymerase chain reaction (RT-PCR) for SARS-CoV-2 between January 2022 and September 2022. The patients were receiving active therapy for their malignancies or were post-Allo-HSCT but still required calcineurin inhibitor (CNI)/antimetabolite agents.

The inclusion criteria were as follows:

- Patients with underlying malignancies including hematologic malignancies or solid tumors;
- Cancer patients with a confirmed diagnosis of COVID-19 by Real-time polymerase chain reaction (RT-PCR) for SAR-CoV-2;
- Cancer patients who developed mild-to-moderate COVID-19 and received either Molnupiravir or Paxlovid.

Patients were excluded if they had a suspected diagnosis of COVID-19 without confirmatory SARS-CoV-2 RT-PCR results. Vulnerable populations (e.g., children, pregnant women, cognitively impaired subjects) were not included.

Patients were identified from the MD Andersons Cancer Center medical records database by searching for patients with prescriptions for Molnupiravir or Nirmatrelvir/Ritonavir. A total of 80 consecutive cancer patients who received Molnupiravir were matched with 160 patients who received Nirmatrelvir/Ritonavir in a 1:2 ratio. Matching was based on age group (18–64 years vs. ≥65 years) and type of cancer (lung cancer vs. hematologic malignancy vs. other solid tumors), and then a simple random sampling method was used to randomly select 160 matching patients treated with Nirmatrelvir/Ritonavir.

### 2.2. Treatment and Data Collection

The dosages were as follows: 800 mg of Molnupiravir (four capsules) was administered every 12 h for 5 days; 300 mg of Nirmatrelvir (two tablets) and 100 mg of Ritonavir (one tablet) were administered every 12 h for 5 days.

The data collected included clinical characteristics, patient demographics, comorbidities, malignancy history, cancer treatment history, symptomology, radiographic findings, laboratory data, antimicrobial therapy, vaccination status, and post-treatment progression and safety information. The primary outcome was progression to severe disease, as measured by hospitalization and mortality rates 29 days after the start of treatment with the study drug. The secondary outcome measured was rebound, or the return of COVID-19 symptoms, within 2 weeks after the last dose of the study treatment. SARS-CoV-2 rebound was defined as the recurrence of signs or symptoms or a new positive viral test result after initial recovery from COVID-19 post-5-day course. To assess safety, we recorded adverse events related to the study medication. We also tracked how many patients had to stop taking other medications because of potential drug–drug interactions in order to take the COVID-19 antiviral.

The study was conducted in compliance with the principles of the Declaration of Helsinki and was approved by the institutional review board at MD Anderson. A waiver of informed consent was obtained since this study posed no risk to the patients given that no clinical or laboratory interventions were made.

### 2.3. Statistical Methods

As a primary analysis, the rate of progression to severe disease was estimated for each arm and compared using an equivalence test for their difference. Given that an equivalence test is equal to two one-sided non-inferiority tests, the rate difference (Molnupiravir–Nirmatrelvir/Ritonavir) and its confidence intervals at an alpha of 0.05 from each side (equal to a two-sided 90% confidence interval) were calculated. Using an equivalence margin of 10%, the 90% confidence interval was checked to see if it fell within (−10%, 10%). For each of the other outcome analyses, chi-square or Fisher’s exact test were used to compare the two treatments. In addition, patient data on demographics, clinical characteristics, medical history, symptomology, and laboratory tests, as well as adverse events and drug–drug interactions, were also compared between the two treatments. Similarly, chi-square or Fisher’s exact test were used to compare categorical variables, and the Wilcoxon rank sum test was used to compare continuous variables. Last, logistic regression analysis was used to identify independent risk factors for progression to severe disease and to assess the independent impact of the treatment difference on progression. All tests except the equivalence test were two-sided at a significance level of 0.05. Data analyses were performed using SAS version 9.4 (SAS Institute Inc., Cary, NC, USA).

## 3. Results

A total of 240 patients with COVID-19 from our database were selected for inclusion in this study. A total of 80 patients received Molnupiravir and 160 patients received Nirmatrelvir/Ritonavir. The baseline characteristics of the two groups are shown in Table 1. Patients were matched on age (median [range]: 66 years [23–91 years] for Nirmatrelvir/Ritonavir vs. 67 years [27–91 years] for Molnupiravir) and type of cancer (lung cancer [20% vs. 20%], hematologic malignancy [40% vs. 40%], and solid tumors excluding lung cancer [40% vs. 40%]). The two groups also had comparable gender distribution (males: 48% for Nirmatrelvir/Ritonavir vs. 54% for Molnupiravir).

In addition to demographics, the two groups were also comparable regarding most comorbidities (Table 1). However, a smoking history (including former and current smokers) was more common in the Molnupiravir group than in the Nirmatrelvir/Ritonavir group (30% vs. 11%, respectively; *p* < 0.001). Similarly, underlying chronic kidney disease (33% vs. 14%; *p* < 0.001) and coronary artery disease (15% vs. 6%; *p* = 0.02) were also more common in the Molnupiravir group. Most of the presenting symptoms were comparable between the two groups except the following factors, which showed significant differences: dyspnea was more prevalent in the Molnupiravir group (15% vs. 6%; *p* = 0.03), and sore throat was more prevalent in the Nirmatrelvir/Ritonavir group (31% vs. 19%; *p* = 0.04) (Table 1). The two groups were comparable in vaccination status, whether fully vaccinated (81% for Nirmatrelvir/Ritonavir vs. 74% for Molnupiravir), partially vaccinated (3% vs. 5%), or not vaccinated (16% vs. 21%).

For the primary outcome of progression to severe disease, the progression rates were 2.6% for patients treated with Nirmatrelvir/Ritonavir and 6.9% for those treated with Molnupiravir. Using an equivalence margin of 10%, the equivalence test showed a rate difference of 4.3% with a 90% confidence interval of (−1.1%, 9.7%), which is within the equivalence interval (−10%, 10%). Therefore, we concluded that the progression rate of Molnupiravir was equivalent to that of Nirmatrelvir/Ritonavir. In addition, we used chi-square or Fisher’s exact test to compare outcomes and no significant difference was found in other clinical outcomes, including rebound of symptoms after treatment, between the two groups (Table 2). There was also no significant difference between the groups in adverse events (4% for Nirmatrelvir/Ritonavir vs. 0% for Molnupiravir, *p* = 0.18). However, patients who received Nirmatrelvir/Ritonavir were more likely than those who received Molnupiravir to have adverse events or have stopped another medication before starting the COVID-19 antiviral to avoid drug–drug interactions (30% vs. 0%, *p* < 0.0001).

For patients who were admitted to hospital for COVID-19, the rates of fever within 24 h of admission, saturation of peripheral oxygen (SpO_2_) at admission, and type of pneumonia were comparable in the two groups (Table 3), as well as absolute neutrophil count, absolute lymphocyte count, and levels of lactate dehydrogenase, C-reactive protein, and procalcitonin. No significant difference between groups was observed for any symptom after hospital admission (Table 3).

Multivariable logistic regression analysis identified two independent risk factors for progression to severe disease: the comorbidity of hypertension (OR = 3.58; 95% CI: 1.50–11.58; *p* = 0.002) and the symptom of cough (OR = 3.00; 95% CI: 1.06–20.45; *p* = 0.035). After adjusting for these factors, no significant treatment difference was observed between Nirmatrelvir/Ritonavir and Molnupiravir in progression to severe disease (*p* = 0.70), which was consistent with the result of the equivalence test (Table 4).

## 4. Discussion

In this retrospective study, we examined the effectiveness of the oral antivirals Nirmatrelvir/Ritonavir and Molnupiravir in the treatment of mild-to-moderate COVID-19 in an outpatient setting in cancer patients at high risk of progression receiving their treatment at MD Anderson. Our study’s findings reveal that in the treatment of mild-to-moderate COVID-19 among cancer patients with hematologic malignancy or solid tumors, Molnupiravir and Nirmatrelvir/Ritonavir exhibited equivalent efficacy in terms of preventing progression to severe disease and mortality. Moreover, Molnupiravir showed a superior safety profile as measured by the incidence of drug–drug interactions and adverse events.

According to the guidelines set forth by the National Institutes of Health, the preferred therapeutic approach for non-hospitalized adults with mild-to-moderate COVID-19 who do not require supplemental oxygen is to initiate treatment with Nirmatrelvir/Ritonavir. Alternatively, if antivirals are needed, recommended treatments include, in order of preference, Remdesivir followed by Molnupiravir [30]. These guidelines, however, were not based on a randomized comparable trial for the two agents; the guidelines were based on the results of two studies that included different populations and control groups.

Several recent studies have compared antivirals in patients with COVID-19 who were considered to be at risk of progression to severe disease. A retrospective cohort study performed in Greece compared patients who took either drug to populations that did not receive therapy and showed that both drugs resulted in a significant reduction in the risk of hospitalization and mortality. This risk reduction was more pronounced in vulnerable populations such as the elderly, the immunocompromised, and those who were unvaccinated [31]. Another retrospective observational study performed in Japan also compared the outcomes of COVID-19 following treatment with either Molnupiravir or Nirmatrelvir/Ritonavir. Severe outcomes were defined as COVID-19-related hospitalization or mortality within 28 days of symptom onset. They concluded that although “the Molnupiravir group had poorer performance status, was older, and had more comorbidities than the Nirmatrelvir/Ritonavir group; nevertheless, the rates of hospitalization, death, and adverse events were similar between the two groups” [32]. Similarly, the retrospective study performed in Europe via the EPICOVIDEHA registry found no statistical difference between the two drugs and yielded results that support the use of Molnupiravir in patients with hematological malignancies [22].

A non-randomized prospective study conducted in Korea evaluated the rates of hospitalization, mortality, and adverse events within 28 days of oral antiviral prescription and found that the rates of hospitalization and death were low and not significantly different between the two arms. Although the prevalence of comorbidities was higher in the Molnupiravir group, adverse events were significantly more frequent in the cohort which received Nirmatrelvir/Ritonavir [33]. Similarly, a prospective study performed in Italy showed that the risk of COVID-19 progression was not statistically different in high-risk patients treated with Nirmatrelvir-Ritonavir, Molnupiravir or a 3-day course of Remdesivir [34].

The data obtained from our research underscore the potential of both medications to effectively combat viral infection in this specific patient population. The parity in effectiveness between Molnupiravir and Nirmatrelvir/Ritonavir provides valuable insights for clinicians and healthcare providers, offering them additional options when tailoring treatment regimens for COVID-19 to individuals with cancer. Our study results are also consistent with those of multiple studies published in the literature; a non-matched observational prospective study in a population at high risk of COVID-19 progression also concluded that there was no difference in efficacy between Nirmatrelvir/Ritonavir and Molnupiravir [34]. In our study, both antivirals have shown comparable ability to reduce progression to severe disease as measured by COVID-19-related hospitalizations and mortality. A similar retrospective study in non-cancer patients echoed our results, showing insignificant differences in post-treatment COVID-19 hospitalizations with 2.8% in the Molnupiravir treatment arm and 3.5% in the Nirmatrelvir/Ritonavir arm (*p*-value = 0.978) and in mortality with 0.4% in the Molnupiravir treatment arm and 3.5% in the Nirmatrelvir/Ritonavir arm (*p*-value = 0.104), respectively [32].

Regarding their safety profiles, our study also showed that Molnupiravir exhibited a superior safety profile compared to Nirmatrelvir/Ritonavir. Nirmatrelvir/Ritonavir has exhibited multiple drug–drug interactions, which is problematic as it requires that other essential medications be discontinued to administer the antiviral. This was of great concern, as many of the physicians at our institute had to discontinue vital drugs in order to administer Nirmatrelvir/Ritonavir in compliance with the guidelines. This finding holds significant clinical relevance, especially in the context of treating cancer patients who need to receive various chemotherapies and accompanying medications to combat their primary disease and related complications. Nirmatrelvir/Ritonavir administration is not always feasible in such populations, as drugs such as tacrolimus, antifungals, and anticoagulants often cannot be stopped or withheld. On the other hand, no such drug–drug interactions exist for Molnupiravir. This concern has also been consistent among all other studies exploring the safety profiles of these two drugs [8,9,10,11].

Patients with cancer have heightened vulnerability to medication side effects; their chemotherapy regimens often cause gastrointestinal side effects that may be exacerbated by the administration of antivirals. Multiple studies have reported on the severe gastrointestinal disturbances seen with Nirmatrelvir/Ritonavir intake. The prospective observational study conducted in Italy documented that 21.1% of patients experienced general adverse events in the Molnupiravir group in comparison to 49.2% in the Nirmatrelvir/Ritonavir arm (*p*-value ≤ 0.001). The most significant difference was observed for dysgeusia, which was experienced by 2.8% of the Molnupiravir group and 41.9% of the Nirmatrelvir/Ritonavir group (*p*-value ≤ 0.001) [34]. These results were obtained in a non-cancer population; we would expect even higher numbers in a cancer population if followed prospectively. Although Molnupiravir has had several side effects reported in the literature, none were severe enough to be reported by patients in our study. Thus, with both medications exhibiting similar efficacy in combating COVID-19, the favorable safety profile of Molnupiravir positions it as a potentially preferable option in cases where minimizing adverse events is paramount. This conclusion underscores the importance of weighing both efficacy and safety considerations when tailoring treatment regimens for COVID-19 in cancer patients.

Our study is one of the largest retrospective studies following the effects of the two drugs in a cancer patient population; however, some limitations affected our study. The main limitation was the retrospective design of this study; our patients were not prospectively followed, hence, some data pertaining to adverse events may be lacking or subject to record bias. Another limitation was that the prescription was given to the patients, but study drug administration could not be verified. We reported the information that was available in our electronic medical health record with the assumption that the patient had received the medication if not otherwise stated in the chart. Finally, prescribing physicians were following NIH guidelines for the treatment of mild-to-moderate non-hospitalized COVID-19 patients, which may have introduced a confounding bias in the selection of patients who were prescribed Molnupiravir.

To our knowledge, this is the first retrospective cohort study that compares the antivirals Nirmatrelvir/Ritonavir and Molnupiravir in a matched immunocompromised cancer patient population in the United States. Further research and real-world prospective evidence are crucial to solidify these findings and guide informed clinical decision-making.

## 5. Conclusions

In the treatment of mild-to-moderate COVID-19 in cancer patients, Molnupiravir was comparable to Nirmatrelvir/Ritonavir in preventing progression to severe disease/death and had a superior safety profile. A prospective randomized study is warranted in this patient population to validate our findings.

## Figures and Tables

**Table 1:** Comparison of characteristics of patients treated with Nirmatrelvir/Ritonavir vs. those treated with Molnupiravir (N = 240).

Characteristics	Nirmatrelvir/Ritonavir	Molnupiravir	*p*-Value
	(n = 160)	(n = 80)	
	N (%)	N (%)	
Age, median (range), years	66 (23–91)	67 (27–91)	0.32
Sex, male	76 (48)	43 (54)	0.36
Race/ethnicity			0.39
White	113/159 (71)	58 (73)	
Black	11/159 (7)	10 (13)	
Hispanic	16/159 (10)	4 (5)	
Asian	18/159 (11)	8 (10)	
Other	1/159 (1)	0 (0)	
Unknown (declined to answer)	1		
Type of cancer			>0.99
Lung cancer	32 (20)	16 (20)	
Hematological malignancy	64 (40)	32 (40)	
Solid tumor excluding lung cancer	64 (40)	32 (40)	
Active cancer therapy within 30 days	103 (64)	50 (63)	0.78
Smoker			<0.001
No	142 (89)	56 (70)	
Current	8 (5)	4 (5)	
Former	10 (6)	20 (25)	
Comorbidities			
Chronic kidney disease	22 (14)	26 (33)	<0.001
Asthma	12 (8)	5 (6)	0.72
Chronic obstructive pulmonary disease	8 (5)	9 (11)	0.08
Congestive heart failure	10 (6)	9 (11)	0.18
Diabetes mellitus	34 (21)	22 (28)	0.28
Coronary artery disease	9 (6)	12 (15)	0.02
Obstructive sleep apnea	9 (6)	7 (9)	0.36
Hypertension	32 (20)	23 (29)	0.14
Venous thromboembolism	17 (11)	13 (16)	0.21
Obesity	41 (26)	19/79 (24)	0.79
Symptoms			
Gastrointestinal symptoms	17 (11)	13 (16)	0.21
Ageusia	7 (4)	5 (6)	0.54
Anosmia	3 (2)	2 (3)	>0.99
Fever/chills	59 (37)	29 (36)	0.92
Dyspnea	10 (6)	12 (15)	0.03
Congestion/rhinorrhea	62 (39)	30 (38)	0.85
Headache/pain	31 (19)	15 (19)	0.91
Fatigue	80 (50)	31 (39)	0.10
Sore throat	50 (31)	15 (19)	0.04
Cough	72 (45)	46 (58)	0.07
COVID-19 vaccination status			0.35
Unvaccinated	26 (16)	17 (21)	
Partially vaccinated	4 (3)	4 (5)	
Fully vaccinated	130 (81)	59 (74)	
Days from last vaccination to COVID-19 diagnosis, median (IQR)	257 (156–413)	318 (237–441)	0.024

Abbreviation: IQR—interquartile range.

**Table 2:** Comparison of outcomes of patients treated with Nirmatrelvir/Ritonavir vs. those treated with Molnupiravir (N = 240).

Outcomes	Nirmatrelvir/Ritonavir	Molnupiravir	*p*-Value
	(n = 160)	(n = 80)	
	N (%)	N (%)	
Antiviral treatment compliance			>0.99
Completed 5 doses	155/159 (97)	77/79 (97)	
Not completed 5 doses	4/159 (3)	2/79 (3)	
Unknown	1	1	
Drug–drug interactions (were any drugs stopped to take the COVID-19 medication?)	44 (28)	0 (0)	<0.0001
30-Day hospital admission	25 (16)	17 (21)	0.28
Admission for COVID-19	12 (8)	11 (14)	0.12
30-Day mortality	0 (0)	2 (3)	0.11
Progression to severe disease *	4/152 (3)	5/72 (7)	0.15
Rebound of symptoms after treatment	5 (3)	7 (9)	0.11
Adverse events	6 (4)	0 (0)	0.18
Drug–drug interactions or adverse events	48 (30)	0 (0)	<0.0001

* Progression to severe disease was defined as either a hospital admission for COVID-19 or death from 6 to 29 days after the start of therapy. Patients who had a hospital admission or died within 5 days after the start of therapy were excluded from this analysis.

**Table 3:** Comparison of symptoms and outcomes by treatment among patients admitted to hospital for COVID-19 (N = 23).

Characteristics	Nirmatrelvir/Ritonavir	Molnupiravir	*p*-Value
	(n = 12)	(n = 11)	
	N (%)	N (%)	
Fever within 24 h of admission	6 (50)	3 (27)	0.40
SpO_2_ at admission, median (range)	97 (90–100)	95 (92–100)	0.34
Pneumonia	4 (33)	4 (36)	>0.99
Type of pneumonia			
Ground-glass opacity	1/4 (25)	1/4 (25)	
Others	3/4 (75)	3/4 (75)	
Side of pneumonia			
Unilateral	1/4 (25)	1/4 (25)	
Bilateral	3/4 (75)	3/4 (75)	
C-reactive protein, median (IQR)	41.8 (18.9–152.7)	11.1 (0.8–37.1)	0.24
Lactate dehydrogenase, median (IQR)	430 (173–492)	224 (174–353)	0.35
Procalcitonin, median (IQR)	0.30 (0.12–1.08)	0.14 (0.11–7.8)	0.94
Absolute neutrophil count, median (IQR)	3.18 (1.27–5.18)	4.08 (1.55–7.22)	0.86
Absolute lymphocyte count, median (IQR)	0.50 (0.33–0.73)	0.42 (0.27–0.47)	0.27
Symptom return	5 (42)	6 (55)	0.54
Symptoms			
Gastrointestinal symptoms	0 (0)	3 (27)	0.09
Ageusia	0 (0)	0 (0)	
Anosmia	0 (0)	0 (0)	
Fever	5 (42)	4 (36)	>0.99
Dyspnea	3 (25)	4 (36)	0.67
Congestion/rhinorrhea	1 (8)	2 (18)	0.59
Headache	1 (8)	0 (0)	>0.99
Fatigue	3 (25)	4 (36)	0.67
Sore throat	1 (8)	1 (9)	>0.99
Cough	3 (25)	5 (45)	0.40
Desaturation (saturation < 94%)	1 (8)	3 (27)	0.32
O_2_ supplement	3 (25)	3 (27)	>0.99
Intubation	0 (0)	0 (0)	
Intensive care unit admission	0 (0)	2 (18)	0.22

Abbreviation: SpO_2_—saturation of peripheral oxygen; IQR—interquartile range.

**Table 4:** Multivariable logistic regression analysis of independent risk factors for progression to severe disease and the independent impact of treatment type (Molnupiravir vs. Nirmatrelvir/Ritonavir) on progression.

Variables	aOR	95% CI	*p*-Value
Hypertension	3.58	1.50–11.58	0.002
Cough	3.00	1.06–20.45	0.035
Type of treatment			0.70
Molnupiravir	1.31	0.56–3.14	
Nirmatrelvir/Ritonavir	Reference		

Abbreviations: aOR—adjusted odds ratio; CI—confidence interval.

## Data Availability

The study protocol, statistical analysis plan, lists of de-identified individual data, and generated tables and figures will be made available upon request by qualified scientific and medical researchers for legitimate research purposes. Requests should be sent to rhachem@mdanderson.org and yijiang@mdanderson.org. Data will be made available on request for 6 months from the date of publication. Investigators are invited to submit study proposal requests detailing research questions and hypotheses in order to receive access to these data.
